# Training of binocular rivalry suppression suggests stimulus-specific plasticity in monocular and binocular visual areas

**DOI:** 10.1038/srep25753

**Published:** 2016-05-10

**Authors:** Mark Vergeer, Johan Wagemans, Raymond van Ee

**Affiliations:** 1 Laboratory of Experimental Psychology, Brain & Cognition, KU Leuven, Belgium; 2 Donders Institute, Radboud University, Department of Biophysics, Nijmegen, The Netherlands; 3 Philips Research Laboratories, Eindhoven, The Netherlands

## Abstract

The plasticity of the human brain, as shown in perceptual learning, is generally reflected by improved task performance after training. Here, we show that perceptual suppression can be increased through training. In the first experiment, binocular rivalry suppression of a specific orientation was trained, leading to a relative reduction in sensitivity to the trained orientation. In a second experiment, two orthogonal orientations were suppressed in alternating training blocks, in the left and right eye, respectively. This double-training procedure lead to reduced sensitivity for the orientation that was suppression-trained in each specific eye, implying that training of feature suppression is specific for the eye in which the oriented grating was presented during training. Results of a control experiment indicate that the obtained effects are indeed due to suppression during training, instead of being merely due to the repetitive presentation of the oriented gratings. Visual plasticity is essential for a person’s visual development. The finding that plasticity can result in increased perceptual suppression reported here may prove to be significant in understanding human visual development. It emphasizes that for stable vision, not only the enhancement of relevant signals is crucial, but also the reliable and stable suppression of (task) irrelevant signals.

The impressive visual abilities of primates can to a large extent be attributed to the plasticity of the brain, which occurs predominantly during early visual development^1^ but continues throughout life, even after the brain’s full maturation^2^,^3^. This plasticity has been exposed experimentally through effects of perceptual learning, where task performance generally enhances after training^2^,^3^. Perceptual learning has been shown for a variety of visual skills, including contrast sensitivity^4^, motion discrimination^5^, orientation discrimination^6^, texture discrimination^7^, vernier acuity^8^ and contour integration^9^. For all these tasks, training results in an improvement in task performance, at least for the trained task and stimulus features, while transfer to untrained tasks or features is usually limited^2^. Observers are generally aware of the trained stimulus and attending the stimulus can boost learning^10^. However, some recent studies have shown that perceptual learning can actually occur implicitly, without the observer attending the learned features^11^ and even without the observer’s awareness of the stimulus. For example, when a non-perceived grating is paired with a reward only when it has a specific orientation, observers improve in detection for this orientation, relative to orientations that are not coupled with a reward^12^.

Although the exact mechanisms and neural architecture involved in perceptual learning are still very much under debate (see Sagi^2^ and Sasaki *et al.*^3^, for recent comprehensive reviews on this topic), reported effects of perceptual learning generally have in common that they show an increase in visual sensitivity after training. However, optimal interaction of an individual with the environment requires a complex, dynamic interplay of not only the enhancement of relevant signals but also the suppression of signals that are irrelevant for the current task at hand (i.e., noise). For example, perceptual selection requires the facilitation of one possible percept while at the same time other (less plausible but possibly valid) alternative interpretations of the visual input are suppressed from perception. The latter principle has been studied most frequently by the use of binocular rivalry paradigms. In binocular rivalry, the left and right eye are presented with different, incompatible information at the same retinal region. As a consequence, only one of these images is perceived, while the other image is perceptually suppressed^13^,^14^. This suppression is generally thought to be largely due to inhibitory networks operating between clusters of neurons coding for each of the presented stimuli, respectively. Although binocular rivalry was traditionally thought to take place through reciprocal inhibitory competition between monocular channels, effects of stimulus competition in binocular rivalry^15^,^16^ have challenged the idea that the neural mechanisms involved in rivalry predominantly have their effect in early (monocular) visual areas and resulted in a consensus that multiple levels of visual processing are involved in resolving binocular rivalry^17^,^18^. Nevertheless, it remains unclear at which processing stage(s) the hypothesized inhibitory networks in binocular rivalry are effective.

A few recent studies have shown support for plasticity in binocular rivalry. With their so-called push-pull paradigm, Xu, He, and Ooi successfully reduce sensory eye dominance by training, at the same time, activation of the observers’ weaker eye and suppression of the observers’ dominant eye^19^,^20^. In addition, it was recently shown that prolonged exposure to binocular rivalry leads to reduced exclusivity and more prevalent mixture percepts, which was interpreted as evidence for inhibitory plasticity in binocular rivalry^21^. The present study takes the study of plasticity of the inhibitory networks involved in binocular rivalry to a next level by focusing on stimulus specificity in suppression training and on the level of visual processing at which these stimulus specific effects have their origin. By training suppression of a specific feature in one eye (Experiment 1), the question if stimulus suppression can be trained will be addressed. Subsequently, a double training protocol will be applied by means of which suppression of different features will be trained in different eyes (Experiment 2), enabling us to identify the role of monocular and binocular processing levels in stimulus suppression and the training of it occurs.

## Results and Discussion

### Experiment 1

**Figure 1:**
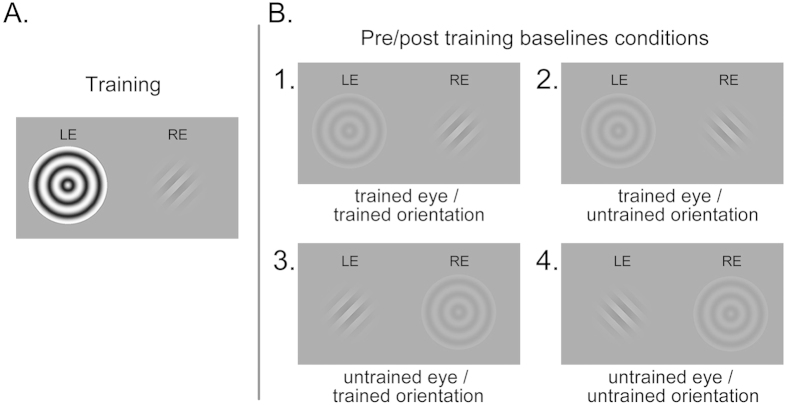
Design of Experiment 1. (**A**) During training, a grating with always the same orientation and presented to the same eye was perceptually suppressed by presenting a high-contrast expanding bull’s eye to the contralateral eye. (**B**) During pre- and post-training baseline sessions, contrast detection thresholds were determined for both orientations, for each eye separately, while at the same time a low-contrast expanding bull’s eye was presented to the contralateral eye to realize binocular rivalry.

In the first experiment, observers were trained to suppress a specific orientation in one eye, while they were unaware of the presentation of the oriented stimulus throughout training, so the suppression training occurred in an implicit manner. During training, in each 1-second trial, an oriented square-wave grating was presented to one eye only, while both the orientation of the grating and the eye to which it was presented remained constant throughout training. The eye and orientation of this oriented grating (either 45 deg or 135 deg) were counterbalanced between observers. A high-contrast, expanding sinusoidal bull’s eye, presented at the corresponding retinal location of the contralateral eye (Fig. 1A), effectuated continuous perceptual suppression of the grating. To help maintain attention to the stimulus location, observers performed a dummy task, for which they were instructed to detect a small contrast change of the bull’s eye that occurred briefly halfway each trial. After finishing the final session of the experiment, observers were debriefed and asked whether they had seen anything else than the bull’s eye during training; all observers responded ‘no’ to this question. When subsequently asked explicitly if they had seen an oriented grating at any point during training, none of the observers responded affirmatively. Training took place in four sessions, each session consisting of four blocks of 160 trials each.

Before and after training, an adaptive QUEST procedure was used to determine contrast detection thresholds for four conditions (Fig. 1B), defined by the eye and orientation of the test grating relative to the trained grating. Here, the expanding bull’s eye was presented at low contrast to allow for perceptual breakthrough of the grating, while binocular rivalry mechanisms were still effective. As during training, the grating and the bull’s eye were both presented for 1 sec. Observers were instructed to manually report if they had seen at least a part of the oriented grating. The target grating was presented in 80% of the trials. The target-present trials were equally distributed according to a 2 × 2 design, where the target was either presented to the left eye or to the right eye, and oriented either 45 degrees to the left or 45 degrees to the right relative to vertical. Within each block, target contrast was varied according to an adaptive QUEST procedure, and 50% detection thresholds were determined, for each target-present condition, separately. Trials of all target-present conditions and the target-absent condition (5 × 50 = 250 trials, in total) were randomly interleaved within each block. This procedure was repeated 3 times and the average of the thresholds from these 3 blocks was taken as pre- and post-training baseline threshold for each condition separately. See the *Experimental Procedures* for a detailed description of the methods.

**Figure 2:**
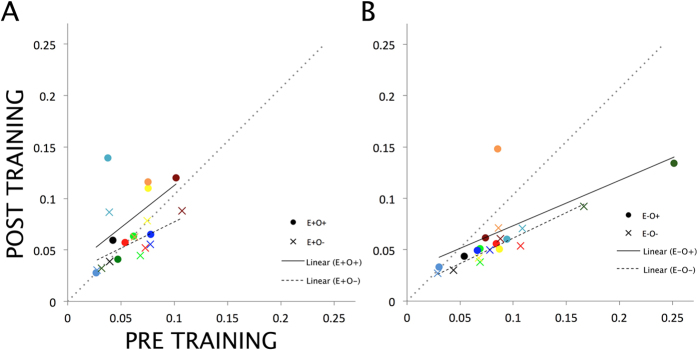
Pre- and post-training contrast detection thresholds. (**A**) Detection thresholds of all 10 observers for the eye in which the grating was presented during training, both for the trained (E + O+) and for the untrained orientation (E + O−). (**B**) Detection thresholds for the same 10 observers, but now for the eye in which the grating was *not* presented during training, again both for the trained (E − O+) and for the untrained orientation (E − O−). Data points are color-coded consistently within observers. Data points below the diagonal dashed line indicate better performance (i.e., lower thresholds) after training than before training, while data points above this line indicate worse performance after training (i.e., higher thresholds). Linear regression lines are added for all conditions.

The pre- and post-training detection thresholds of all observers for all conditions are plotted in Fig. 2. The linear regression lines indicate that for the eye in which the grating was presented during training (panel A), post-training performance compared to pre-training performance is relatively worse for the trained orientation than for the untrained orientation. A similar pattern can be seen for the eye in which no grating was presented during training (panel B).

**Figure 3:**
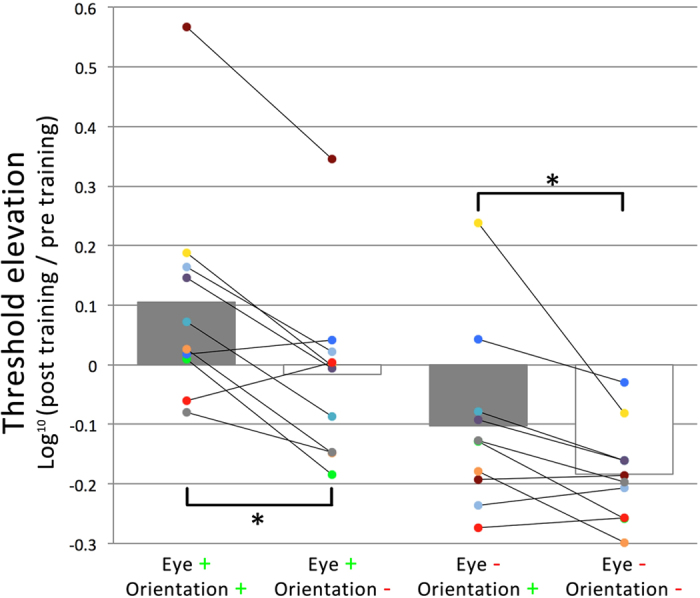
Results of Experiment 1. Log-transformed threshold elevations defined as log^10^ (post-training/pre-training threshold), where negative and positive values correspond with performance increments and decrements in contrast detection after training, respectively. Bars represent group means, while dots represent individual observer data, color-coded consistently within observers.

Analyses were performed on the log-transformed threshold elevations (log^10^ (post-training threshold/pre-training threshold)), which were computed for each condition and for each participant separately (see Fig. 3). The log transformation was executed to adjust for a non-normal distribution in the original threshold elevation data (i.e., by definition, ratios are skewed to the right of the mean, while a log transformation of these ratios effectuate a normal distribution around 0). A repeated-measures ANOVA revealed a significant main effect for eye of presentation (F_1,9_ = 10.0, *p* < 0.05) and a significant main effect of grating orientation (F_1,9_ = 15.7, *p* < 0.005), but no interaction between eye of presentation and grating orientation. One-sample t-tests show that performance improved significantly only when the target was presented in a different eye and with a different orientation compared to training (*t*_9_ = 7.12, *p* < 0.001). Subsequently, pairwise t-tests were performed to test the effect of orientation for each eye separately. For the eye in which the grating was presented during training, threshold elevation was significantly higher for the *trained* orientation than for the *untrained* orientation (*t*_9_ = 3.97, *p* < 0.005). This was also the case for the contralateral eye (*t*_9_ = 2.55, *p* < 0.05). These data indicate that suppression can be increased through implicit training, as reflected by impaired detection for the eye and orientation that were trained to be suppressed, relative to the opposite eye and orientation, respectively. The transfer of the orientation-selective suppression effect to the contralateral eye suggests the involvement of binocular visual areas in this orientation suppression effect. Although higher level effects in binocular rivalry^15^,^16^ have led to the suggestion that inhibitory interactions in binocular visual areas might be involved in resolving binocular rivalry^17^, binocular rivalry suppression is generally thought to predominantly rely on inhibitory competition between monocular channels^22^. Therefore, there was no clear a priori prediction regarding cross-eye transfer of any potential orientation effect. The improved performance found for the untrained orientation in the eye in which no grating was presented during training most likely reflects an eye-based learning effect, as the training protocol biases vision in favor of the dominant eye during training (i.e., the eye in which the bull’s eye was presented), in line with recent findings on ocular dominance training^19^,^20^. Note that here ‘eye-based learning’ refers to an improvement in performance, whereas the term ‘suppression learning’ is used consistently throughout the manuscript to refer to a relative performance decrement as the result of training. A second experiment was conducted to explicitly disentangle monocular and binocular contributions in suppression learning effects as found in Experiment 1.

### Experiment 2

**Figure 4:**
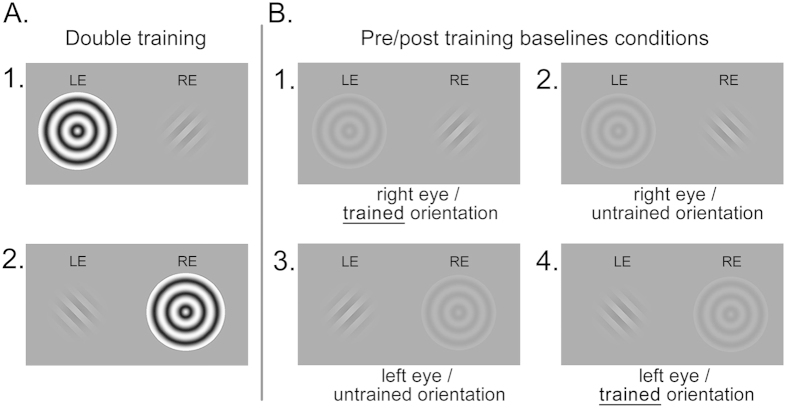
Design of Experiment 2. (**A**) In half of the training blocks, observers were trained on suppressing one orientation presented to one eye, while in the other half they were trained on suppressing the orthogonal orientation presented to the other eye. (**B**) During pre- and post-training baseline sessions, contrast detection thresholds were determined for both orientations, for each eye separately, while at the same time a low-contrast expanding bull’s eye was presented to the contralateral eye to realize binocular rivalry.

In Experiment 2, a similar paradigm was used as in Experiment 1, but now observers were subjected to an implicit, double-training protocol (Fig. 4A). This double training aimed at disentangling suppression learning effects occurring at a monocular processing stage from effects occurring at a binocular stage of processing. In half of the training trials, a grating with one orientation was suppressed in one eye, while in the other half gratings with the orthogonal orientation were suppressed in the other eye. Hence, suppression of both orientations was trained equally at a binocular level of processing. Therefore, if suppression learning merely has a binocular origin, then the double-training protocol in Experiment should cancel out any orientation-specific differences in the learning pattern. The total number of training trials was doubled relative to Experiment 1, to keep training per orientation equal in both experiments. The eye in which either orientation was trained was counterbalanced between observers. Baseline conditions (Fig. 4B) were the same as in Experiment 1, but could now be defined as either the same or different, relative to the trained orientation for the respective eye.

**Figure 5:**
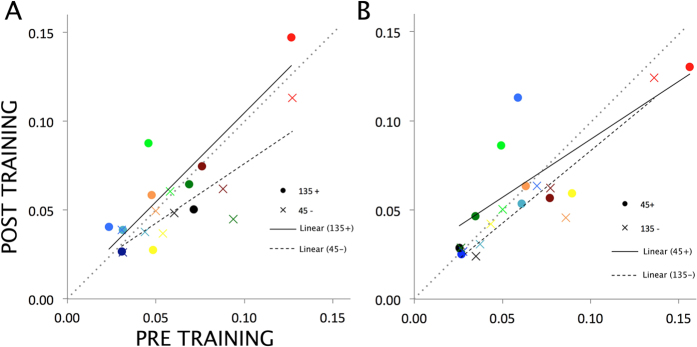
Pre- and post-training contrast detection thresholds. (**A**) Detection thresholds of all 10 observers for the eye in which the 135deg oriented grating was presented in half of the trials during training, both for the trained (135+) and for the untrained orientation (45−). (**B**) Detection thresholds for the same 10 observers, but now for the eye in which in the other half of the training trials the 45deg oriented grating was presented during training, again both for the trained (45+) and for the untrained orientation (135−). Data points are color-coded consistently within observers. Data points below the diagonal dashed line indicate better performance (i.e., lower thresholds) after training than before training, while data points above this line indicate worse performance after training (i.e., higher thresholds). Linear regression lines are added for all conditions.

Pre- and post-training contrast detection thresholds for both orientations and for each eye separately are presented in Fig. 5 for all ten observers. A first look at these data reveals that for each eye, post-training performance compared to pre-training performance is relatively worse for the orientation that was presented during training, relative to the orientation that was not presented in that respective eye during training.

**Figure 6:**
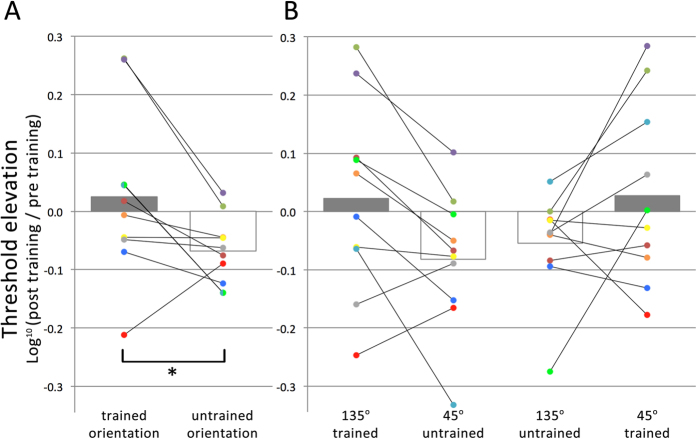
Results of Experiment 2. (**A**) Log-transformed threshold elevations from pre-training to post-training collapsed over both eyes averaged across all 10 observers. (**B**) Each observer was trained on suppressing a 135 degrees oriented grating in one eye and a 45 degrees oriented grating in the other eye. Here the effects of training are shown for each eye separately, with the trained orientation in one eye being the untrained orientation in the other eye. In both panels, bars represent group means, while dots represent individual observer data, color-coded consistently within observers.

Threshold elevations were computed for each observer and for each condition separately, and analyses were performed on the log-transformed threshold elevations (see Fig. 6). Comparing pre-training contrast detection thresholds with post-training threshold, collapsed over both eyes, a significant overall effect of orientation is obtained, where threshold elevation is significantly higher for the orientation that was trained in a certain eye, relative to the untrained orientation (*t*_9_ = 2.48; *p* < 0.05). For the untrained orientation there was a significant improvement after training (*t*_9_ = 3.71, *p* < 0.005), while no significant improvement or deterioration in performance was found for the trained orientation. When looking at the trained orientations separately (Fig. 3B), the effect of orientation was significant only for the eye in which the 135° oriented grating was trained (*t*_9_ = 2.73; *p* < 0.05).

These data show effects of suppression learning selective for the eye for which the suppression of an orientation was trained. This suggest that the training of stimulus suppression is effective already during monocular stages of visual processing, as indicated by the differential effects of suppression learning found for trained and untrained orientations. Suppression of both orientations has been trained equally at a binocular level of processing. Therefore, if suppression learning would merely have had a binocular origin, then the double-training protocol in Experiment 2 should have cancelled out any orientation-specific differences in the learning pattern. However, note that the effect size of orientation suppression is significantly lower here as compared to Experiment 1, which supports the claim that both monocular and binocular processing stages are involved in the effects of orientation suppression learning that are presented here.

### Control Experiment

A control experiment was performed to test whether the effects reported in Experiment 1 and 2 were indeed effects of perceptual suppression instead of being merely due to adaptation. The experimental paradigm was to a large extent similar as the paradigm used in Experiment 2. The pre- and post-training baseline conditions were identical to those in both of the main experiments. Presentation of the oriented grating during training followed a similar protocol as in Experiment 2, with the main difference that here this grating was no longer perceptually suppressed by stimulation (i.e., presentation of the bull’s eye) in the contralateral eye. In other words, the presented grating was visible throughout the full training period. During training, gratings were slightly tilted relative to the reference (i.e., 45deg for gratings presented to one eye in half of the blocks, and 135deg for gratings presented to the other eye in the other half of the blocks), and observers performed an orientation discrimination task on the presented gratings following an adaptive QUEST procedure. The protocol of grating presentation was similar to the double training protocol used in Experiment 2, with the same numbers of trials and blocks per eye and orientation. The average orientation discrimination threshold across blocks and observers was 1.81 degrees (SD = 1.41 degrees). The effect of training was quantified by computing the linear regression slope on the orientation discrimination thresholds of the 32 training blocks per observer separately (Mean = −0.039). A paired-samples t-test revealed that the effect of training was not significant across observers.

**Figure 7:**
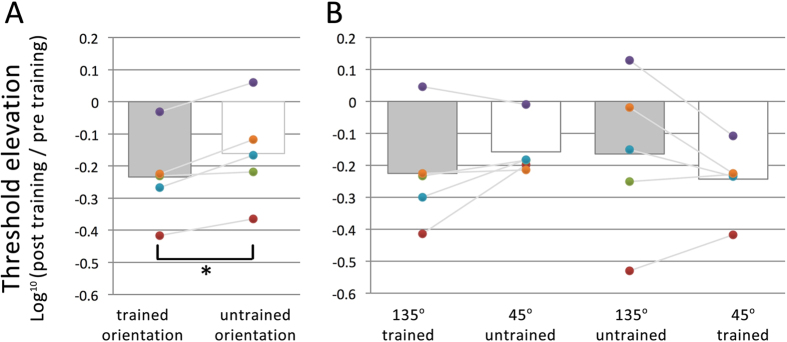
Results of the Control experiment. (**A**) Log-transformed threshold elevations from pre-training to post-training collapsed over both eyes averaged across all 5 observers. (**B**) Here the effects of training on contrast detection are shown for each eye separately, with the trained orientation in one eye being the untrained orientation in the other eye. In both panels, bars represent group means, while dots represent individual observer data, color-coded consistently within observers.

Comparison of pre- and post-training thresholds per baseline condition reveals an opposite pattern as compared to Experiment 2 (see Fig. 7). Here, log-transformed threshold elevation for all five observers is lower for the trained orientation than for the untrained orientation, indicating that mere (visible) exposure leads to improved detection performance specific for the eye in which the grating is presented (*t*_5_ = 4.08, *p* < 0.05). This result further strengthens our claim that the learning effects observed in the main experiments are indeed effects of suppression training, instead of being due to the build-up of adaptation during training for instance.

## General Discussion

Perceptual learning research commonly shows enhanced performance after training, reflecting an increase in *visibility* of the trained feature. We show here that the perceptual *suppression* of unseen representations can be increased through training. As suppression is commonly largely attributed to cross-inhibition between competing neural populations, we argue that the strengthening of inhibitory networks through training is the most plausible mechanism responsible for the effects reported here. This is in line with recent neurophysiological evidence on inhibitory plasticity, suggesting that Hebbian style learning principles also apply to inhibitory neural circuits^23^. It was recently shown that GABAergic inhibition reduces as a result of short-term monocular deprivation^24^. This reduction of GABA concentration was shown to be associated with a boost in perceptual performance in the stimulation-deprived eye. In addition, it was recently shown that GABA decreases the rate of perceptual switches in bistable perception, in case of motion-induced blindness and structure from motion^25^. It can be argued that this behavioral effect results from an increase in mutual inhibition between competing stimulus-specific neural populations due to an increase in GABA. Accordingly, training perceptual suppression might lead to an increase in GABA concentration. However, the question whether effects of GABA are variable enough at a fine scale to account for the feature specific effects that we report here remains speculative.

It has already been shown that there is increased suppression of task-irrelevant features after motion-discrimination learning^26^, but this effect is related to attention and occurred only when the irrelevant features were presented above visibility threshold during training^27^. Following a similar line of reasoning it has recently been argued that effects of implicit, or task irrelevant learning do not occur when the irrelevant signal is sufficiently noticeable to be picked up by the attentional system^3^. In that case this signal is suppressed, which prevents learning to occur. However, in case of task irrelevant perceptual learning, the irrelevant signals are not picked up and suppressed by the attentional system and, hence, they are not reinforced through feedback (reward) on the primary task. In a similar way, in our training task on the dominant stimulus, the reinforcement signal may have enhanced not only the dominant stimulus signal but also the inhibitory signal responsible for suppressing the grating in the contralateral eye.

The precise mechanisms of binocular rivalry suppression and the responsible cortical areas are still under debate. A widely held view is that suppression, to a large extent, occurs in a non-selective way, where the input of the non-dominant eye is simply not reinforced, for instance due to the absence of attention to these stimuli^25^. Recent binocular rivalry studies have shown that binocular rivalry suppression of a stimulus might actually lead to a selective reduction in the sensitivity of the suppressed stimulus features^28^,^29^. Stimulus-specific behavioral effects in binocular rivalry in general are often considered to be supportive of the involvement of binocular visual areas in binocular rivalry^16^. Current theories on binocular rivalry suggest that competitive processes in both monocular and binocular visual areas are involved in binocular rivalry^17^,^30^. However, the idea that binocular rivalry suppression involves not only inhibitory competition at a monocular level but also inhibitory competition between binocular stimulus representations is still under debate^31^. It was recently suggested that monocular areas are involved in stimulus rivalry^32^. By using an implicit learning paradigm, we support this claim and provide further evidence that both monocular and binocular processing areas are involved in stimulus specific effects in binocular rivalry.

The findings presented here may also prove to be important for the plasticity-stability dilemma that the brain faces. Successful interaction with our surroundings requires on the one hand plasticity of the system to be able to adapt to ever changing circumstances, but on the other hand a certain degree of stability, where the state of the system does not flip with every small change in input^3^. To achieve this, the plasticity of mechanisms responsible for perceptual suppression that we show here may prove to be of significant importance, as the suppression of (task-)irrelevant input (i.e., noise) contributes to the relative enhancement of the currently relevant visual input (i.e., by increasing signal-to-noise ratio).

In sum, we show that perceptual suppression can be trained in an eye and stimulus specific fashion, indicating inhibitory plasticity at a monocular stage of processing and supporting a monocular account of stimulus rivalry. The intricate interplay between perceptual plasticity (excitatory neural interactions) and perceptual stability (inhibitory interactions) in binocular vision enabled us to infer that observers can learn to perceptually suppress specific features (such as orientation) of visual stimulation at a processing level already before sensory inputs of both eyes have been integrated.

## Methods

### Experiment 1

#### Observers

Ten naïve observers (all female) participated in the experiment. They all had normal or corrected-to-normal vision. All observers were paid or received course credits for their participation, and they all gave informed consent at the beginning of the experiment. The ethical committee of the Faculty of Psychology and Educational Sciences of the University of Leuven approved the experiment and the experiment was conducted in accordance with the committee’s guidelines.

#### Apparatus

Stimuli were shown on a 22” Dell LCD display (1920 × 1080 at 60 Hz) driven by a Dell Optiplex 755 PC running on Windows 7. A 4-mirror stereo setup achieved binocular presentation. The left eye and right eye image were presented on the left and right side of the screen, respectively. A vertically oriented black cardboard splitter was positioned between the horizontal center of the monitor and the stereo setup to avoid parts of the images being visible to the contralateral eye. A head and chin rest positioned at 4 cm from the mirrors were used to stabilize head position and orientation. The effective viewing distance was 180 cm. Stimulus presentation, timing and keyboard responses were controlled with custom software programmed in Python 2.7 using the PsychoPy library^33^,^34^.

#### Stimuli

To maintain stable vergence throughout the experiment, four black lines were continuously presented to each eye at corresponding retinal locations (above, below, left, and right of the stimulus, respectively). These so-called vergence lines (with dimensions 0.51 × 0.05 arcdeg were presented with a distance of 1.57 arcdeg between stimulus center and the center of each vergence line. During training, a square-wave grating (size = 2.15 arcdeg, spatial frequency = 2.42 cycles/arcdeg and contrast = 0.2, filtered by a Gaussian) was presented at the fovea of one eye. The orientation of this grating was either 45° of 135° from vertical. In the contralateral eye, a sinusoidal expanding bull’s eye was presented (size = 2.48 arcdeg, radial spatial frequency = 2.42 cycles/arcdeg, radial speed = 0.99 cycles/arcdeg, contrast = 0.8).

The stimuli used for the baseline measurements were largely similar to those used during training. The grating was presented with a variable contrast following an adaptive QUEST procedure with a starting value of 0.2. In addition, the bull’s eye was presented at a constant low contrast (varied between observers between 0.02–0.06), to allow for rivalry between the bull’s eye and the grating. It was by default set to 0.06. However, with this contrast a few observers were incapable of perceiving any gratings presented to one eye, even at full grating contrast, due to strong eye dominance. Therefore, for these observers, we lowered the bull’s eye contrast to a level for which contrast detection thresholds could be determined for both eyes.

#### Design and procedure

Preceding the first experimental session, the positioning of the right and left eye vergence lines were spatially calibrated, for each observer individually, to ensure binocular stimulus presentation at corresponding retinal locations. In the baseline sessions (sessions 1 and 6, pre- and post-training, respectively), contrast detection thresholds were measured for 4 conditions. In each trial, the low-contrast expanding bull’s eye was presented foveally, either to the left or to the right eye. In the contralateral eye a target grating was presented in 80% of the trials. The target-present trials were equally distributed according to a 2 × 2 design, where the target was either presented to the left eye or to the right eye, and oriented either 45 degrees to the left or 45 degrees to the right relative to vertical. The task for the observers was to indicate for each trial if a target was present or not. Within each block, target contrast was varied according to an adaptive QUEST procedure, and 50% detection thresholds were determined, for each target-present condition, separately. From the beginning of each 1-second trial, the target contrast was ramped up from 0 to its current QUEST-value with a speed of 180%/second. Trials of all target present conditions and the target absent condition (5 × 50 = 250 trials, in total) were randomly interleaved within each block. This procedure was repeated 3 times and the average of the thresholds from these 3 blocks was taken as pre- and post-training baseline threshold for each condition separately. The training sessions (sessions 2–5) consisted of 4 blocks of 160 trials each, leading to a total of 2560 training trials. All 6 sessions took place within one week, between Monday and Friday, with the restrictions of no more than 2 sessions per day and at least 1 hour break between each 2 training sessions, while the last training session and the post-training baseline measurement took place at 2 consecutive days.

During training, a high-contrast expanding bull’s eye was presented to one eye, while a grating (contrast = 0.2) was presented to the contralateral eye. The grating was ramped-up in contrast in a similar fashion, and with the same speed, as in the baseline sessions. Because of the high saliency of the expanding bull’s eye, the grating was perceptually suppressed throughout the whole training stage. Upon debriefing after the full experiment, none of the observers indicated that they had seen any of the presented oriented gratings at any moment during training. Within an observer, the grating was presented in the same eye, and with the same orientation in each trial. The eye and the orientation at which the suppressed grating was presented were counterbalanced between observers. During training, the task for observers was to detect a small, brief contrast change occurring for the visible bull’s eye midway each trial. This task was included to make sure that observers maintained attention to the stimulus.

### Experiment 2

#### Observers

Ten naïve observers (8 female) participated in the experiment. They all had normal or corrected-to-normal vision. All observers were paid or received course credits for their participation, and they all gave informed consent at the beginning of the experiment. The ethical committee of the Faculty of Psychology and Educational Sciences of the University of Leuven approved the experiment and the experiment was conducted in accordance with the committee’s guidelines.

#### Apparatus

The same setup was used as for Experiment 1.

#### Stimuli

The stimuli and individual trials were the same as in Experiment 1.

#### Design and procedure

Pre- and post-training baseline contrast detection thresholds measurements were identical to Experiment 1. Where observers were trained on suppressing a single orientation in one eye in Experiment 1, observers were subjected to a double-training protocol in Experiment 2, which led to a doubling of the total amount of training trials per session. Each training session consisted of 8 blocks of 160 trials each. In half of these blocks observers were trained on suppressing a 45 degrees oriented grating presented in one eye, while in the other half of blocks they were trained on suppressing a 135 degrees oriented grating presented to the other eye. In each training session, 4 blocks of suppressing one of the two orientations were followed by 4 blocks of training the orthogonal orientation. Which orientation was trained in which eye and the order of trained orientations within a session were counterbalanced between observers.

### Control Experiment

#### Observers

Five naïve observers (4 female) participated in the experiment. They all had normal or corrected-to-normal vision. All observers received course credits for their participation, and they all gave informed consent at the beginning of the experiment. The ethical committee of the Faculty of Psychology and Educational Sciences of the University of Leuven approved the experiment and the experiment was conducted in accordance with the committee’s guidelines.

#### Apparatus

The same setup was used as for Experiment 1 and Experiment 2.

#### Stimuli

The stimuli were similar as in Experiment 1 and 2.

#### Design and procedure

Pre- and post-training baseline contrast detection thresholds measurements were identical to Experiment 1 and Experiment 2. Observers were trained on an orientation discrimination task, according to a double training protocol. Each training session consisted of 8 blocks of 160 trials each, similar as in Experiment 2. In half of these blocks, observers were trained to discriminate the orientation of a grating relative to 45 degrees in one eye, while in the other half of blocks they were trained to discriminate the orientation of the grating relative to 135 degrees presented to the other eye, in both cases following adaptive QUEST procedures. Observers were instructed to indicate whether a grating was tilted towards horizontal or towards vertical. In each training session, 4 blocks of one condition (either 45deg or 135deg) were followed by 4 blocks of training the orthogonal orientation, but now presented to the contralateral eye. Which orientation was trained in which eye and the order of trained orientations within a session were counterbalanced between observers. The starting tilt of the grating relative to the reference orientation was 5 degrees.

## Additional Information

**How to cite this article**: Vergeer, M. *et al.* Training of binocular rivalry suppression suggests stimulus-specific plasticity in monocular and binocular visual areas. *Sci. Rep.* **6**, 25753; doi: 10.1038/srep25753 (2016).

## References

[b1] Gibson E. J. Principles of perceptual learning and development. (Appleton-Century-Crofts, 1969), Ch. 1, 1–18.

[b2] Sagi D. Perceptual learning in Vision Research. Vision Res. 51, 1552–1566 (2011). 20974167 10.1016/j.visres.2010.10.019

[b3] Sasaki Y., Nanez J. E. & Watanabe T. Advances in visual perceptual learning and plasticity. Nat. Rev. Neurosci. 11, 53–60 (2010). 19953104 10.1038/nrn2737 PMC2864603

[b4] De Valois K. K. Spatial frequency adaptation can enhance contrast sensitivity. Vision Res. 17, 1057–1065 (1977). 595415 10.1016/0042-6989(77)90010-4

[b5] Ball K. & Sekuler R. A specific and enduring improvement in visual motion discrimination. Science 218, 697–698 (1982). 7134968 10.1126/science.7134968

[b6] Vogels R. & Orban G. A. The effect of practice on the oblique effect in line orientation judgments. Vision Res. 25, 1679–1687 (1985). 3832592 10.1016/0042-6989(85)90140-3

[b7] Karni A. & Sagi D. Where practice makes perfect in texture discrimination: evidence for primary visual cortex plasticity. Proc. Natl. Acad. Sci. 88, 4966–4970 (1991). 2052578 10.1073/pnas.88.11.4966 PMC51788

[b8] Mckee S. P. & Westhe G. Improvement in vernier acuity with practice. Percept. Psychophys. 24, 258–262 (1978). 704286 10.3758/bf03206097

[b9] Gilbert C. D., Li W. & Piech V. Perceptual learning and adult cortical plasticity. J. Physiol. 587, 2743–2751 (2009). 19525560 10.1113/jphysiol.2009.171488 PMC2718234

[b10] Ahissar M. & Hochstein S. Attentional control of early perceptual learning. Proc. Natl. Acad. Sci. 90, 5718–5722 (1993). 8516322 10.1073/pnas.90.12.5718 PMC46793

[b11] Watanabe T., Náñez J. E. & Sasaki Y. Perceptual learning without perception. Nature 413, 844–848 (2001). 11677607 10.1038/35101601

[b12] Seitz A. R., Kim D. & Watanabe T. Rewards evoke learning of unconsciously processed visual stimuli in adult humans. Neuron 61, 700–707 (2009). 19285467 10.1016/j.neuron.2009.01.016 PMC2683263

[b13] Wheatstone C. Contributions to the Physiology of Vision. Part the First. On Some Remarkable, and Hitherto Unobserved, Phenomena of Binocular Vision. Philos. Trans. R. Soc. Lond. 128, 371–394 (1838).

[b14] Blake R. & Wilson H. Binocular vision. Vision Res. 51, 754–770 (2011). 20951722 10.1016/j.visres.2010.10.009 PMC3050089

[b15] Kovács I., Papathomas T. V., Yang M. & Fehér Á. When the brain changes its mind: Interocular grouping during binocular rivalry. Proc. Natl. Acad. Sci. 93, 15508–15511 (1996). 8986842 10.1073/pnas.93.26.15508 PMC26435

[b16] Logothetis N. K., Leopold D. A. & Sheinberg D. L. What is rivalling during binocular rivalry? Nature 380, 621–624 (1996). 8602261 10.1038/380621a0

[b17] Tong F., Meng M. & Blake R. Neural bases of binocular rivalry. Trends Cogn. Sci. 10, 502–511 (2006). 16997612 10.1016/j.tics.2006.09.003

[b18] Wilson H. R. Computational evidence for a rivalry hierarchy in vision. Proc. Natl. Acad. Sci. 100, 14499–14503 (2003). 14612564 10.1073/pnas.2333622100 PMC283620

[b19] Xu J. P., He Z. J. & Ooi T. L. Push-pull training reduces foveal sensory eye dominance within the early visual channels. Vision Res. 61, 48–59 (2012). 21689673 10.1016/j.visres.2011.06.005 PMC3202681

[b20] Xu J. P., He Z. J. & Ooi T. L. Effectively Reducing Sensory Eye Dominance with a Push-Pull Perceptual Learning Protocol. Curr. Biol. 20, 1864–1868 (2010). 20951044 10.1016/j.cub.2010.09.043 PMC2963665

[b21] Klink P. C., Brascamp J. W., Blake R. & van Wezel R. J. A. Experience-driven plasticity in binocular vision. Curr. Biol. 20, 1464–1469 (2010). 20674360 10.1016/j.cub.2010.06.057 PMC2926173

[b22] Blake R. A neural theory of binocular rivalry. Psychol. Rev. 96, 145–167 (1989). 2648445 10.1037/0033-295x.96.1.145

[b23] Hooser S. D. V., Escobar G. M., Maffei A. & Miller P. Emerging feed-forward inhibition allows the robust formation of direction selectivity in the developing ferret visual cortex. J. Neurophysiol. 111 2355–73 (2014). 24598528 10.1152/jn.00891.2013 PMC4099478

[b24] Lunghi C., Emir U. E., Morrone M. C. & Bridge H. Short-Term Monocular Deprivation Alters GABA in the Adult Human Visual Cortex. Curr. Biol. 25, 1496–1501 (2015). 26004760 10.1016/j.cub.2015.04.021 PMC5040500

[b25] Vidnyánszky Z. & Sohn W. Learning to suppress task-irrelevant visual stimuli with attention. Vision Res. 45, 677–685 (2005). 15639494 10.1016/j.visres.2004.10.009

[b26] Paffen C. L. E., Verstraten F. A. J. & Vidnyánszky Z. Attention-based perceptual learning increases binocular rivalry suppression of irrelevant visual features. J. Vis. 8, 25, 1–11 (2008). 18484864 10.1167/8.4.25

[b27] van Loon A. M. *et al.* GABA shapes the dynamics of bistable perception. Curr. Biol. 23, 823–827 (2013). 23602476 10.1016/j.cub.2013.03.067

[b28] Stuit S. M., Cass J., Paffen C. L. E. & Alais D. Orientation-tuned suppression in binocular rivalry reveals general and specific components of rivalry suppression. J. Vis. 9, 1–15 (2009). 10.1167/9.11.17 20053080

[b29] Vergeer M. & van Lier R. Feature-based activation and suppression during binocular rivalry. Vision Res. 50, 743–749 (2010). 20102726 10.1016/j.visres.2010.01.011

[b30] Blake R. & Logothetis N. K. Visual competition. Nat. Rev. Neurosci. 3, 13–21 (2002). 11823801 10.1038/nrn701

[b31] Brascamp J., Sohn H., Lee S.-H. & Blake R. A monocular contribution to stimulus rivalry. Proc. Natl. Acad. Sci. 110, 8337–8344 (2013). 23610414 10.1073/pnas.1305393110 PMC3666721

[b32] Lee S.-H. & Blake R. A fresh look at interocular grouping during binocular rivalry. Vision Res. 44, 983–991 (2004). 15031091 10.1016/j.visres.2003.12.007

[b33] Peirce J. W. PsychoPy—Psychophysics software in Python. J. Neurosci. Methods 162, 8–13 (2007). 17254636 10.1016/j.jneumeth.2006.11.017 PMC2018741

[b34] Peirce J. W. Generating stimuli for neuroscience using PsychoPy. Front. Neuroinformatics 2, 1–8 (2009). 10.3389/neuro.11.010.2008 PMC2636899 19198666

